# Use of mandarin and persimmon fruits in water kefir fermentation

**DOI:** 10.1002/fsn3.3561

**Published:** 2023-08-11

**Authors:** Zeynep B. Güzel‐Seydim, Gülçin Şatır, Çağlar Gökırmaklı

**Affiliations:** ^1^ Department of Food Engineering Süleyman Demirel University Isparta Turkey; ^2^ Department of Nutrition and Dietetics Süleyman Demirel University Isparta Turkey

**Keywords:** antioxidant activity, fermentation, mandarin, persimmon, water kefir grains

## Abstract

Water kefir is a non‐dairy probiotic beverage. It is obtained by fermentation of water kefir grains with a sugar solution. This study aims to determine the properties of water kefir beverages obtained by fermentation of mandarin and persimmon‐containing water for 42 h. According to microbiological results, both fruits containing water samples provided a high number of lactic acid bacteria and yeasts. Moreover, after fermentation, pH, Brix, and dry matter content did not significantly differ. On the other hand, fructose, maltose, and acetic acid contents of mandarin water kefir are significantly higher than persimmon water kefir (*p* < .05). Persimmon water kefir had higher total phenolic contents, twice as much as mandarin water kefir (*p* < .05). Both water kefirs had good color properties. The organoleptic acceptability of the fruit water kefirs was promising.

## Graphical abstract

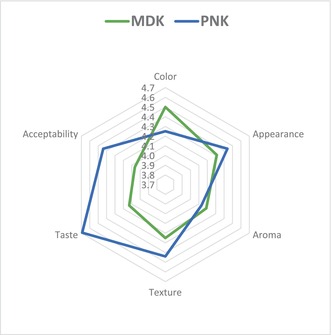

## 1 INTRODUCTION

Consumers are increasingly concerned about incorporating healthy foods into their diets, increasing the demand for probiotics, prebiotics, and functional fermented beverages. Water kefir is produced by fermentation of sugar solution with water kefir grains containing lactic acid bacteria such as *Lactobacillus paracasei*, *Lactobacillus casei*, *Lactobacillus hilgardii*, *Lactobacillus nagelii*, and yeasts like *S. cerevisiae* (Gökırmaklı & Güzel‐Seydim, 2022). With potential health benefits, water kefir is gaining popularity as a fermented food using non‐dairy substrates like vegetables, fresh/dried fruits, and plant‐based milk. Thus, it also stands out as an important source of probiotics for both vegans and allergic individuals. Water kefir is a refreshing fermented product with slight CO_2_ and has an acidic, slightly fruity taste and flavor. Water kefir has antimicrobial, anti‐inflammatory, antioxidant, hepato‐protective, anti‐hyperglycemic, anti‐diabetic, and IBS prophylaxis properties (Aspiras et al., 2015; Gökırmaklı et al., 2023; Hsieh et al., 2012; Koh et al., 2018).

Fermentation conditions such as inoculation rate of water kefir grains, temperature and length, type and concentration of sugar used, and mineral content of the water are essential for water kefir fermentation. However, fermentation of fruits with water kefir grains would affect the characteristics of the final product due to the extraction of important nutrients from the fruits, such as sugar, vitamins, and minerals, for the growth of the microbiota. The nutritional composition is directly related to the type and concentration of the fruits, which influence the product's characteristics (Laureys et al., 2018, 2019). Furthermore, fruit kefirs could provide superior organoleptic properties (Bueno et al., 2021; Farag et al., 2020; Gökırmaklı et al., 2023; Tu et al., 2019).

Bioactive components are abundant in fresh fruits (Kaur et al., 2023; Rodríguez et al., 2021). Mandarin and persimmon, in particular, contain phenolic substances ranging from 100 to 457 mg/L (Kaur et al., 2022; Saini et al., 2022). The identification of flavonoids with anticarcinogenic effects in studies has increased the demand for fruits containing antioxidants and carotenoids. Mandarin (*Citrus reticulata*) is one of these fruit species because of its health benefits. On the other hand, Citrus fruits can be used to produce functional foods and maintain viability for probiotic microorganisms (Cesur, 2014; Sendra et al., 2008). Persimmon (*Diospyros kaki*) is rich in nutrients and bioactive components such as proteins, sugar, vitamin A, vitamin B_6_, vitamin B_12_, vitamin D, vitamin C, vitamin E, calcium, potassium, polyphenols, flavonoids, and carotenoids (Pachisia, 2020). The need for continuous use of the rich nutrient‐dense persimmon fruit throughout the year while accounting for post‐harvest and transportation losses, adding value to the food production chain, and contributing to food sustainability is also crucial for the economy (Kaur et al., 2022). Fermentation of fruit‐added water with water kefir grains could be an alternative added‐value preservation technique (Hur et al., 2014).

Fruits‐ and vegetables‐based water kefir productions have been investigated. Fruit juices are considered good nutrients for the growth of water kefir microorganisms, presenting nutrients that favor microbial growth. Probiotic drink was made by fermentation of grape juice with water kefir microorganisms as a healthy beverage alternative (Santos et al., 2023). Furthermore, water kefir is considered a potential prebiotic, probiotic, and antioxidant source for vegans and individuals who are intolerant/allergic to dairy products. Randazzo et al. (2016) developed non‐dairy beverages from various fruits such as apple, quince, kiwifruit, grape, pomegranate, and prickly pear with the usage of water kefir microorganisms. Corona et al. (2016) also produced kefir‐like beverages by fermenting vegetable juices (carrot, melon, fennel, tomato, onion, and strawberry) with water kefir grains. These studies indicated the potential for developing value‐added and functional fruit‐ or vegetable‐based kefir‐like beverages.

There are some studies on water kefir beverages (Baú et al., 2013; Corona et al., 2016; Cui et al., 2013; Du & Myracle, 2018; Fiorda et al., 2016; Koh et al., 2018; Łopusiewicz et al., 2019; Puerari et al., 2012; Randazzo et al., 2016; Rodrigues et al., 2016); however, there is no study on the fermentation of persimmon‐ and mandarin‐based water kefirs. This study aimed to investigate the effects of the fruits, *Citrus reticulata* and *Diospyros kaki* on the water kefir's microbial, physicochemical, and sensory properties.

## 2 MATERIALS AND METHODS

### 2.1 Materials

Mandarin (*Citrus reticulata*) and dried Persimmon (*Diospyros kaki*) were purchased from a local market (Antalya, Turkey) on March 2021. Water kefir grains were obtained from Danem Dairy Products (Isparta, Turkey).

### 2.2 Production of fruit water kefir

**FIGURE 1:**
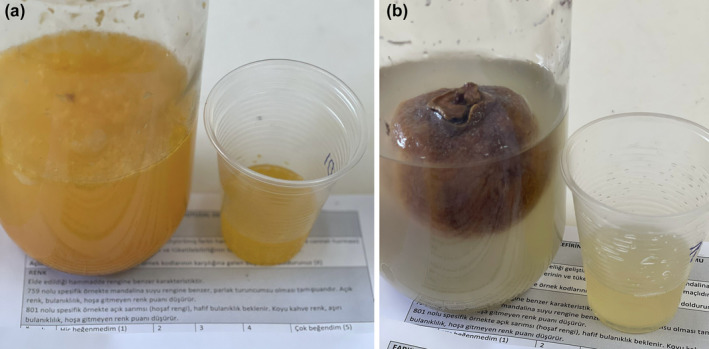
The mandarin (a) and persimmon (b) of water kefirs.

Mandarin juices were obtained from mandarin fruits. 250 mL mandarin juice was mixed with 500 mL sterile water for mandarin‐based water kefir production. 250 g dried persimmon was treated in boiling water for 2 min. Then, persimmon was taken and added to 750 mL (25°C) sterile water. Water kefir grains were added to prepared mandarin and persimmon samples at a rate of 2% (w/w). Fermentation was carried out at 25°C for 42 h. During the fermentation, pH was measured and recorded (Figure 1). After completion of fermentation, mandarin (MDK)‐ and persimmon (PNK)‐based water kefir samples were stored at 4°C for a day, and then, the analyses were carried out.

#### 2.2.1 Microbiological analyses

One milliliter of each sample was diluted with 9 mL of sterile peptone water. Samples were appropriately diluted and plated using MRS Agar (Merck), M17 Agar (Merck), and PDA (Merck) to enumerate *Lactobacillus* spp.*, Lactococcus* spp., and yeasts, respectively. A standard incubation temperature appropriate for each target microorganism was used. Microbiological analysis results were expressed as log CFU mL^−1^ (Spencer & de Spencer, 2008).

### 2.3 Physicochemical analysis

Total soluble solid contents (Brix) were carried out at 20°C using an Abbe refractometer (Bellingham Stanley Limit 60/70 Refractometer, UK). pH was obtained by directly measuring fruit‐based water kefir samples using a pH meter (Seven Compact, Mettler Toledo, USA) equipped with a glass electrode. pH measurement was done in duplicate, and the experiment was conducted in triplicate during fermentation.

### 2.4 Determination of sugar profile

Sugar contents (fructose, glucose, maltose, and sucrose) were determined according to the HPLC method of Petkova and Denev (2013) with some modifications. A one‐gram sample was extracted with 8 mL of hot water (60–65°C) for 15 min (frequently stirring to aid sugar dissolution), as well as 1 mL of both Carrez 1 (15 g potassium hexacyanoferrate in 100 mL of water) and Carrez 2 (30 g of zinc sulfate in 100 mL of water). The supernatant was poured into a flask, and the residue was extracted twice with 5 mL of hot water (80°C). The extract (1 mL) was filtered through an Oasis HLB cartridge preconditioned with 1 mL of methanol and 1 mL of HPLC‐grade water to clean it. The first eight drops of eluent were collected and transferred to an HPLC vial. The HPLC system used the RI detector, and separations were performed on a SUPELCOSIL LC‐NH_2_ column (Supelco, Dorset, UK) at 30°C at a rate of 1.0 mL/min. The injection volume was 20 μL. The solvent employed was a 75:25 mixture of acetonitrile and HPLC‐grade water. The identified sugars were quantified using peak areas and a calibration curve with corresponding standards.

### 2.5 Organic acids

The organic acid measurement was performed according to Guzel‐Seydim et al. (2000) with slight modification. Extraction was done with 10 g of each water kefir sample, 1.4 M acetic acid, and 0.3 M metaphosphoric acid solution. One gram of sample and 25 mL of ethanol were used to make an ethanol extract, which was then processed through a 0.45 μm filter with an ultrasonicator for 1 h. Chromatographic analyses were conducted on an Agilent 1200 HPLC system with an SPD‐10Avp UV–VIS detector operating at 275 nm. An isocratic elution with a mobile‐phase H_3_PO_4_/H_2_O (pH 2.2) at a 0.8 mL/min flow rate was used. The column used was a TR‐016059 Tracer Extrasil ODS2 supplied by Tecknokroma S. Coop. C. Ltd. (Barcelona, Spain). Organic acid quantification was based on calibration curves created for each of the compounds identified in the samples. Standard organic acids included acetic acid, citric acid, formic acid, fumaric acid, lactic acid, malic acid, oxalic acid, and tartaric acid.

### 2.6 Total phenolic content (GAE)

The Folin–Ciocalteu colorimetric method, described by Singleton et al. (1999), was used to determine the total phenolic content of the beverages. Gallic acid was used as the standard phenolic compound. Briefly, 50 μL of the filtered extracts were mixed with 450 μL of distilled water and 2.5 mL of 0.2 N Folin–Ciocalteu reagent. The absorbance of the resulting blue‐colored solution was measured at 765 nm after incubation at 30°C for 1.5 h with intermittent shaking. Quantitative measurements were performed, based on a standard calibration curve of six points of gallic acid in 80% methanol. The total phenolic content was expressed as gallic acid equivalents (GAE) in mg/L. The results were expressed as mg of gallic acid equivalent (GAE) per 100 mL of fruit water kefir samples (*y* = 5.5629*x* + 0.249: *R* ^2^ = 0.9753).

### 2.7 Trolox equivalent antioxidant capacity (TEAC) 2,2‐Azinobis (3‐ethylbenzthiazoline)‐6 sulfonic acid assay

This method was conducted according to Re et al. (1999). Briefly, ABTS^+^ radical cation is generated by reacting 7 mM ABTS^+^ and 2.45 mM potassium persulfate via incubation at room temperature in the dark for 12–16 h. The ABTS^+^ solution was diluted with 80% HPLC‐grade ethanol to an absorbance of 0.700 ± 0.040 at 734 nm and equilibrated at 30°C. After introduction of a 30 μL aliquot of each dilution into the assay, it produced from 20 to 80% inhibition of the blank absorbance. To 3 mL of diluted ABTS^+^, 30 μL of each sample was added and mixed thoroughly. The reactive mixture was allowed to stand at room temperature for 6 min, and the absorbance was recorded immediately at 734 nm. Trolox standard solutions (concentrations from 0 to 2.5 mM) in 80% ethanol were prepared and assayed using the same conditions. Appropriate solvent blanks were run in each assay. The percent of inhibition of absorbance at 734 nm was calculated and plotted as a function of concentration of Trolox for the standard reference data. The absorbance of the resulting oxidized solution was compared to that of the calibrated Trolox standard. The absorbance was recorded at 734 nm. TEAC results have been expressed as mM Trolox equivalents (*y* = 83.877*x* + 30.485: *R* ^2^ = 0.9989).

### 2.8 Radical scavenging activity using DPPH (2.2‐diphenyl‐1‐picrylhydrazyl) method

Radical scavenging activity of the water kefir was determined as described by Singh et al. (2002). Different concentrations of fruit water kefir samples and BHA (25, 50, and 100 ppm) were taken. DPPH was diluted to 3 mL with ethanol for samples for free radical scavenging activity. Then, 1 mL of prepared DPPH solution (1 mM) was added to the samples and mixed thoroughly with the help of vortex and left for 30 min of incubation at 30°C in the dark. The samples' absorbance changes were measured at 517 nm (Shimadzu UV‐1800, Japan). The inhibition percentage of radical scavenging activity was calculated using the following formula: % radical scavenging activity = (Control OD = sample OD/Control OD) × 100.

### 2.9 Color analysis

Minolta Chroma Meter (CR‐400; Konica Minolta, Inc.) was used to determine the color of both fruit water kefir samples. CIE The color space coordinates L* (lightness), a* (red‐green), and b* (yellow‐blue) were recorded using a white standard calibration plate (*Y* = 92.7, *x* = 0.3160, *y* = 0.3321) (Sogut & Seydim, 2023). The glass light projection tube (Minolta, CR‐A33e) was used to take five measurements on each sample. Experiments were carried out with two replications. The results were presented as the mean standard deviation.

### 2.10 Descriptive sensory evaluation

Five main and 16 sub‐sensory quality parameters were used to assess the sensory quality of fruit water kefirs (appearance, aroma, taste, texture, and acceptability). A group of eight trained panelists (five men and three women ranging in age from 25 to 52 years) conducted the tests to identify five descriptors based on the samples' appearance, taste, aroma, texture, and perception. A sensory panel was assembled, and the organoleptic assessment was graded using a five‐point scale, with five being highly desirable and one being highly undesirable. Fruit water kefir samples were served in opaque plastic cups at 7–10°C and coded with three‐digit numbers.

### 2.11 Statistical analysis

Descriptive statistics (percentages, means, and standard deviations) were used to determine the sample characteristics of three replicates. The paired t‐test was used to assess differences in beverage components. SPSS v24.0 was used (IBM). The significance level was set at 0.05.

## 3 RESULTS AND DISCUSSION

### 3.1 Microbial content

After fermentation with water kefir grains, both fruit‐based kefir samples had similarly high *Lactobacilli* spp. and *Lactococci* spp. The *lactococci* and *lactobacilli* counts of mandarin and persimmon kefir reached 7.87–7.84 log CFU mL^−1^ and 8.08–8.16 log CFU mL^−1^, respectively (Table 1). Based on the microbial analyses, mandarin and persimmon without added sugar have provided an optimum nutritive content for the growth of the lactic acid bacteria. Previous studies have also shown that water kefir containing Russian olive fruit (*Elaeagnus angustifolia*), apple, quince, grape, kiwi fruit, prickly pear, pumpkin, red pitaya, and pomegranate could be efficient carriers of lactic acid bacteria (Darvishzadeh et al., 2021; Bueno et al., 2021; Randazzo et al., 2016; Ozcelik et al., 2021; Koh et al., 2018). The presence of yeast in fruit‐based water kefir is related to the yeast content of water kefir grain microbiota. Fruits provided optimal nutrients for yeast growth as a substrate (Table 1). Fruit‐based water kefir would provide a diverse range of healthy microbiota (Pendón et al., 2022). Water extraction of sugar in the fruits promoted yeast growth and viability in water kefirs. According to Gökırmaklı and Güzel‐Seydim (2022), water kefir grains contain a wide range of microbiota that would provide a variety of bioactive compounds obtained during fermentation, including partial nutrient degradation.

**TABLE 1:** Microbiological contents of fruit‐based water kefir samples (log CFU/mL).

	MDK	PNK
*Lactobacillus* sp.	8.08 ± 0.15	8.16 ± 0.14
*Lactococcus* sp.	7.87 ± 0.16	7.84 ± 0.01
Yeasts	7.59 ± 0.06^a^	6.17 ± 0.56^b^

*Note*: The different superscript letters in the same row show a significant difference between the kefir grains (*p* < .05). Results were expressed as mean ± standard deviation.

Abbreviations: MDK, Mandarin‐based kefir, PNK, Persimmon‐based kefir.

### 3.2 Proximate composition

The acidification observed during fermentation and storage is caused primarily by LABs, AABs, and yeasts consuming mainly sugars present in the substrate. After the fermentation, pH of the MDK and PNK were 4.00 and 4.07, respectively (Table 2). The pH significantly decreased during persimmon‐based kefir fermentation from 6.02 (0 h) to 4.07 (42 h). Fruits with high acidity, such as grape juice, provide a favorable medium for the growth of yeasts. In the study, increasing the water kefir grain proportions in grape juice caused a slight pH variation from 2.94 to 2.89 (Santos et al., 2023).

**TABLE 2:** Chemical analysis.

	MDK	PNK
pH	4.00 ± 0.02	4.07 ± 0.03
^°^Brix	2.10 ± 0.05^a^	3.00 ± 0.00^b^
Dry matter (%)	2.23 ± 0.17^a^	4.31 ± 0.25^b^

*Note*: The different superscript letters in the same row show a significant difference between the kefir grains (*p* < .05). Results were expressed as mean ± standard deviation.

Abbreviations: MDK, Mandarin‐based kefir; PNK, Persimmon‐based kefir.

^°^Brix values of both fruits added water samples were 4^°^ before the fermentation started. During the fermentation, due to the sugar usage of microbiota, the ^°^Brix values of the samples similarly decreased, as shown in Table 2 (*p* > .05). During the fermentation, sugars were converted into ethanol and CO_2_, and thereby the sugar content of water kefir beverages made with fruit juices decreased. Similar results were reported in previous studies for water kefir‐like beverages (Esatbeyoglu et al., 2023; Randazzo et al., 2016).

### 3.3 Sugar profiles of fruit‐based kefirs

The sugar profiles of MNK and PNK are given in Table 3. Fructose, glucose, and maltose were significantly higher in the MDK than in PNK (*p* < .05), while glucose and sucrose contents in MDK and PNK were slightly different (*p* > .05). Sucrose is one of the main carbohydrates during the fermentation processes of water kefir, and it can be metabolized by three different pathways in many LAB species. The sugar profiles found in this study are comparable to those findings by Bueno et al. (2021), Destro et al. (2019), and Puerari et al. (2012). Laureys et al. (2018) reported that sucrose level of 50 g/L was completely consumed after 24 h of fermentation in water kefir. They also indicated that during the first 24 h of fermentation, sucrose decreased. Sugars in both fruit samples were well‐used and significantly decreased during fermentation with water kefir grains due to microbial growth and metabolism. At the end of the fermentation, glucose use of microbiota occurred in water kefir; however, some glucose was left in both kefir samples and provided slight sweetness in the final product. Because microbial findings showed that yeasts were well grown during fermentation, it was thought that yeast in water kefir grains would use this sugar profile better than lactose (Table 3).

**TABLE 3:** Sugar profiles of fruit‐based water kefirs.

g 100 mL^−1^	MDK	PNK
Fructose	4.14 ± 1.20^a^	1.62 ± 0.62^b^
Glucose	3.28 ± 1.12	2.72 ± 1.31
Sucrose	2.63 ± 1.36	1.71 ± 1.09
Maltose	4.44 ± 1.57^a^	2.39 ± 1.24^b^

*Note*: The different superscript letters in the same row show a significant difference between the kefir grains (*p* < .05). Results were expressed as mean ± standard deviation.

Abbreviations: MDK, Mandarin‐based kefir; PNK, Persimmon‐based kefir.

### 3.4 Organic acid contents

Organic acid formation in fermented products is an indicator of microbial metabolic activity. Notably, the acetic acid was higher in mandarin water kefir samples (*p* < .05), whereas the malic acid was higher in persimmon water kefir (Table 4). Various factors, including fruit type, maturity, season, geographic origin, and storage conditions, could influence the composition of organic acid compounds in water kefir made from fruits. Kefirs made using mandarin, and persimmon contained 1.16 and 1.11 g/L lactic acid, respectively. Lactic acid levels significantly increased at the end of the fermentation due to the metabolic activity of the water kefir grain microbiota. Table 3 shows that the microbiota efficiently utilized the sugars in the fruit water kefir samples during fermentation, and metabolites such as organic acids increased, and pH decreased. Water kefir fermentation promotes bacterial metabolism, resulting in acidification, that is, faster conversion of sucrose to its monosaccharides and lactic acid. MDK and PNK samples had acetic acid, whose esters are responsible for water kefir's fruity flavor and aroma; acetic acid was previously identified in water kefir (Randazzo et al., 2016; Zannini et al., 2022).

**TABLE 4:** Organic acid contents of water kefir samples.

Organic acid	MDK (mgL^−1^)	PNK (mg‐L^−1^)
Acetic acid	602.46 ± 25.86^a^	316.29 ± 8.96^b^
Lactic acid	1159.22 ± 41.28	1112.68 ± 31.06
Malic acid	62.14 ± 5.41^a^	218.11 ± 4.08^b^

*Note*: LOD: 0.25 mg/L for citric acid; 0.04 mg/L for oxalic and fumaric acid; 0.12 mg/L for tartaric acid. The different superscript letters in the same row show a significant difference between the kefir grains (*p* < .05). Results were expressed as mean ± standard deviation.

Abbreviations: MDK, Mandarin‐based kefir; PNK, Persimmon‐based kefir.

### 3.5 Antioxidant capacities of fruit‐based kefirs

Results of total phenolic content, TEAC, and DPPH analysis are reported in Table 5. PNK had significantly higher total phenolic content than MNK (*p* < .05). On the other hand, the MDK and PNK had TEAC values of 15.89 and 9.44 mmol TE L^−1^, respectively (*p* < .05); however, DPPH values of both fruit‐based kefirs were similar (*p* > .05). After water kefir fermentation of soy and quinoa protein concentrates, the total phenolic content and antioxidant activity increased significantly (Alrosan et al., 2023; Tu et al., 2019). Fruit‐based fermented kefirs contain antioxidants, which lower the risk of cardiovascular disease, cancer, atherosclerosis, and diabetes (Jideani et al., 2021). Tu et al. (2019) found that after two days of fermentation, the ABTS radical scavenging activity and ferric‐reducing antioxidant power of plant‐based water kefirs significantly increased. In line with our findings, Randazzo et al. (2016) observed a greater antioxidant capacity in water kefirs produced with kiwi and pomegranate (Jideani et al., 2021).

**TABLE 5:** Antioxidant capacities of fruit‐based kefir samples.

	MDK	PNK
GAE (mg/L)	22.34 ± 2.67^a^	49.30 ± 2.36^b^
TEAC (mmol TE L^−1^)	15.89 ± 2.59^a^	9.44 ± 0.31^b^
DPPH (mM)	2.43 ± 0.51	2.29 ± 0.28

*Note*: The different superscript letters in the same row show a significant difference between the kefir grains (*p* < .05). Results were expressed as mean ± standard deviation.

Aronia pomace kefir demonstrated a greater DPPH radical scavenging activity (Esatbeyoglu et al., 2023). Corona et al. (2016) developed water kefir‐like beverages made from vegetable juices, and they observed a decrease in DPPH values apart from melon and tomato. Randazzo et al. (2016) also produced non‐dairy beverages produced from Mediterranean fruit juices and found decreases in DPPH values except quince with slight increase after fermentation. The potent antioxidant activity of mandarin results from phenols, flavonoids, condensed tannins, and ascorbic acid (Bureš et al., 2022). Carotenoids are the primary pigment present in persimmon. They are an excellent source of lipid‐soluble antioxidants, especially lutein, zeaxanthin, and astaxanthin, having the ability to scavenge free radicals in a lipid‐soluble environment and thus preventing the oxidation of lipids (Pachisia, 2020).

### 3.6 Color

Because of its relationship with other physicochemical and sensory properties, color is important for beverages. L*, a*, and b* values of MDK and PNK were reported in Table 6. Undoubtedly, the type and fermentation of water kefir affected the final product's color and organoleptic properties. The MDK had light, relatively shiny yellowish color; on the other hand, the PNK had light brownish color. The MDK had significantly higher lightness values than the PNK (*p* < .05). That subjective evaluation of fruit water kefir samples was compared to the results shown in Table 6; after fermentation, the effect of the process on color was also demonstrated. Regarding color parameters, the browning process during fermentation could explain the decrease in lightness and redness in samples. This phenomenon is due to activating specific oxidases, such as polyphenol oxidase, when the environments are not entirely anaerobic (Corona et al., 2016).

**TABLE 6:** Color values of fruit kefirs.

Attributes	Mandarin	Persimmon
L*	24.99 ± 0.91^a^	19.79 ± 1.13^b^
a*	−0.94 ± 0.06^a^	0.017 ± 0.02^b^
b*	4.86 ± 0.22^a^	0.043 ± 0.01^b^

*Note*: The different superscript letters in the same row show a significant difference between the kefir grains (*p* < .05). Results were expressed as mean ± standard deviation.

The yellowness of the persimmon water kefir samples was higher (Table 6). The negative value of the a* parameter indicated that the mandarin water kefir was reddish, whereas the positive value of the b parameter indicated that it was yellowish. The presence of carotenoid pigments such as β‐carotene in fruit‐based water kefirs could explain the yellowish color. The low a and b values found in this study showed that fermentation would not cause significant changes in the final product's redness or yellowness. The color properties of fruit‐based water kefirs had acceptable fruity‐like colors. Similarly, Corona et al. (2016) found the total color difference in water kefir prepared with carrot and fennel to be 2.94 and 11.55, respectively, and Randazzo et al. (2016) reported the total color difference as 3.41 and 14.91 for water kefir prepared with kiwifruit and prickly pear, respectively.

### 3.7 Sensory evaluation

**FIGURE 2:**
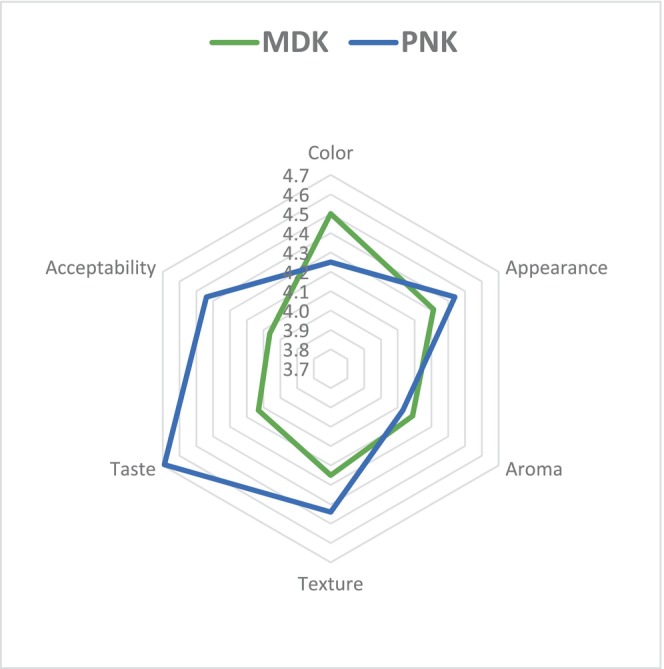
Scores of attributes for sensory evaluation.

In this study, the descriptive words for mandarin and persimmon fruit‐based kefir samples were homogenous texture with bright color, slightly acidic, slightly alcoholic, and presence of CO_2_, yeasty aroma, liquorish flavor, and refreshing taste (Figure 2). Except for color and aroma, the persimmon water kefir samples scored the best for all sensory properties (Table 7). There was a significant difference (*p* < .05) in taste and acceptability attributes for water kefirs based on fruit types. The acceptability of water kefirs was nearly average for participants. The uniformity in color and flavor was attributed to fermentation. Fruity, acidic, and yeasty taste was identified among sub‐sensory descriptors of water kefirs in this study. Based on this context, grape juice with the addition of water kefir obtained a better acceptance by the panelists concerning color, acidity, aroma, and overall impression, obtained mean scores for all evaluated attributes ranging from 6.76 to 8.35 (Santos et al., 2023).

**TABLE 7:** Sensory scores of water kefir samples.

	MNK	PNK
Appearance	4.31 ± 0.21	4.44 ± 0.34
Color	4.50 ± 0.10	4.25 ± 0.11
Aroma	4.19 ± 0.33	4.13 ± 0.29
Taste	4.13 ± 0.57^a^	4.69 ± 0.38^b^
Texture	4.25 ± 0.40	4.44 ± 0.67
Acceptability	4.06 ± 0.82^a^	4.44 ± 0.69^b^

*Note*: The different superscript letters in the same row show a significant difference between the kefir grains (*p* < .05). Results were expressed as mean ± standard deviation.

Abbreviations: MDK, Mandarin‐based kefir; PNK, Persimmon‐based kefir.

It has been stated that the sensory properties of water kefir vary depending on the type and quantity of water kefir culture, fermentation, and storage temperature, the type of fruit or vegetable used in production, and the content of water‐soluble substances (Corona et al., 2016; Puerari et al., 2012; Randazzo et al., 2016).

## 4 CONCLUSION

Studies on the technological and functional properties of water kefir are important because various fruits greatly impact and differ in product characteristics. Also, fermentation of water kefir grains with alternative substrates is needed to extend the beneficial health effects of water kefir for non‐dairy consumers, vegans, and allergic/lactose intolerant individuals. This study obtained water kefir beverages with high antioxidant activity properties using mandarin and persimmon fruits. Moreover, the microbiological contents of fruit‐based water kefir were very satisfying due to the nourishing environment provided by fruits. Even though the sensory scores of the fruit water kefirs were promising, further studies are necessary to optimize their organoleptic properties.

## CONFLICT OF INTEREST STATEMENT

The authors declare that they have no known competing financial interests or personal relationships that could have appeared to influence the work reported in this paper.

## Data Availability

The results/data/figures in this manuscript have not been published elsewhere.
